# 

**Published:** 1900-03-31

**Authors:** 


### NOTICE TO CORRESPONDENTS.

All MS., Letters, Books for Review, and other matters intended for the Editor should be addressed The Editor, "The Hospital," The Hospital Building, 28 & 29, Southampton Street, Strand, W.C.

The Editor cannot undertake to return rejected MS., even when accompanied by stamped directed envelope.

**Notice to Advertisers and Subscribers.**

All Advertisements, Orders for Copies of The Hospital, and Business Communications should be addressed to **THE MANAGER (Not to the Editor),** The Hospital, The Hospital Building, 28 & 29, Southampton Street, Strand, London, W.C.

The Cost of Subscription, post free, is as follows:—Per week, 2½d.; per quarter, 2s. 8d.; per half-year, 5s. 3d.; per annum, 10s. 6d. Foreign:—Per annum, 15s. 2d., payable, in all cases, in advance.

**NOTICE.—Vol. XXVI. of "The Hospital" (April, 1899, to September, 1899), in handsome cloth binding, is now on sale at the Office of this Journal, price 7s. 6d. Cases for binding the Numbers contained in this Volume 1s. 6d. Index to Vol. XXVI., 2½d., post free.**

**The Monthly Part for April will be ready on 2nd April. Price 6d., by post 8d.**

### The Student of Medicine.

### APPOINTMENTS.

R. H. Allport, M.R.C.S., L.R.C.P., appointed Assistant Medical Officer of the Renfrew Road Workhouse of the Parish of St. Mary, Lambeth. P. D. Barker, M.R.C.S., L R.C.P., appointed Senior House Surgeon to the Sheffield Royal Infirmary. W. L. Brown, M A., M.B., B.C.Cantab., M.R.C.P.Lond., appointed Assistant Physician and Pathologist to the Metropolitan Hospital. H. H. A. Carter, L.R.C.P.I., L.R.C.S.I., appointed Assistant Medical Officer in the Colonial Service. A. B. Clarke, L.R.C.S., L.S.A., appointed Medical Officer of the Marland District of Jorrington Union. J. E. Clements, M.B., B.Ch., B.A.O.R.U.I., appointed Medical Officer to the Chinese Customs Service. J. Herbert Dixon, M.B., Mast.-Surg.Edin., appointed Honorary Surgeon to the Birkenhead Women's Hospital.

Royal 8vo., illustrated by a series of Original Photo-micrographs and many Illustrations in the Text, cloth gilt, 7s 6d. net,

THE MENOPAUSE AND ITS DISORDERS.

With Chapters on Menstruation.
By A. D. LEITH NAPIER, M.D., M.R.C.P.
London: The Scientific Press, Ltd., 28 & 29, Southampton Street, Strand, W.C.

### Hovis Bread

(TRADE MARK.)

Makes every meal nicer, and is excellent when toasted.

Most Family Bakers make and deliver Hovis daily. Should your Baker not make it, we shall be pleased to send you the address of your nearest agent. The Hovis Bread Flour Co., Ld., Macclesfield.
Samples sent free to Nurses or Members of the Medical Profession.

### BOOKS FOR NURSES.

| | |
|---|---|
| **Domville's Manual for Hospital Nurses,** 8th edition ... ... ... ... ... ... | **2/6** |
| **Cullingworth's Monthly Nursing,** 4th edition... | **1/6** |
| **Hellier's Gynæcological Nursing...** ... ... | **1/6** |
| **Cuff's Lectures on Medicine to Nurses..** ... | **3/6** |
| **Richmond's Antiseptic Principles for Nurses** | **1/-** |
| **Mayne's Short Dictionary of Medical Terms,** | **2/6** |
| **Chavasse's Advice to a Wife,** 14th edition, by Dr. FANCOURT BARNES ... ... ... ... | **2/6** |
| **Chavasse's Advice to a Mother,** 15th edition, by Dr. GEORGE CARPENTER .. ... ... ... | **2/6** |

LONDON: J. & A. CHURCHILL, 7, Great Marlborough Street.

### By FLORENCE BAXENDALE.

This Day. Crown 8vo., cloth. Price **3/6.**

### THE DISENCHANTMENT OF NURSE DOROTHY.

A Realistic, Faithful, and Striking Story of Hospital and Nursing Life and Routine, As It Actually Exists. Written by one who knows from long practical experience.

SKEFFINGTON AND SON, 163, PICCADILLY, W.

*Publishers to H.M. the Queen, and to H.R.H. the Prince of Wales.*

A REPRINT OF

### A NURSING HANDBOOK

TOGETHER WITH S. MAW, SON, AND THOMPSON'S

### COMPLETE CATALOGUE OF NURSING REQUISITES.

The above work is now republished at a cost of 1s. per copy. Messrs. S. M., S., and T. have been compelled to make this charge on account of the extreme popularity of the work and the great expense incurred in its production.

***Post Free on receipt of 1s.***

### S. MAW, SON, and THOMPSON,

7 to 12, ALDERSGATE STREET, LONDON, E.C.

